# Retinal analysis of a mouse model of Alzheimer’s disease with multicontrast optical coherence tomography

**DOI:** 10.1117/1.NPh.7.1.015006

**Published:** 2020-02-04

**Authors:** Danielle J. Harper, Marco Augustin, Antonia Lichtenegger, Johanna Gesperger, Tanja Himmel, Martina Muck, Conrad W. Merkle, Pablo Eugui, Stefan Kummer, Adelheid Woehrer, Martin Glösmann, Bernhard Baumann

**Affiliations:** a Medical University of Vienna, Center for Medical Physics and Biomedical Engineering, Vienna, Austria; b General Hospital and Medical University of Vienna, Institute of Neurology, Vienna, Austria; c University of Veterinary Medicine, Institute of Pathology, Vienna, Austria; d University of Veterinary Medicine, Core Facility for Research and Technology, Vienna, Austria

**Keywords:** optical coherence tomography, Alzheimer’s disease, retina, polarization, amyloid beta, histology

## Abstract

**Significance.** Recent Alzheimer’s disease (AD) patient studies have focused on retinal analysis, as the retina is the only part of the central nervous system that can be imaged noninvasively by optical methods. However, as this is a relatively new approach, the occurrence and role of retinal pathological features are still debated.

**Aim.** The retina of an APP/PS1 mouse model was investigated using multicontrast optical coherence tomography (OCT) in order to provide a documentation of what was observed in both transgenic and wild-type mice.

**Approach.** Both eyes of 24 APP/PS1 transgenic mice (age: 45 to 104 weeks) and 15 age-matched wild-type littermates were imaged by the custom-built OCT system. At the end of the experiment, retinas and brains were harvested from a subset of the mice (14 transgenic, 7 age-matched control) in order to compare the *in vivo* results to histological analysis and to quantify the cortical amyloid beta plaque load.

**Results.** The system provided a combination of standard reflectivity data, polarization-sensitive data, and OCT angiograms. Qualitative and quantitative information from the resultant OCT images was extracted on retinal layer thickness and structure, presence of hyper-reflective foci, phase retardation abnormalities, and retinal vasculature.

**Conclusions.** Although multicontrast OCT revealed abnormal structural properties and phase retardation signals in the retina of this APP/PS1 mouse model, the observations were very similar in transgenic and control mice.

## 1 Introduction

It is hypothesized that cerebral changes precede Alzheimer’s disease (AD) symptom presentation by over 20 years.^1^ Since the beginning of the millennium, the number of deaths from AD in the United States has increased by 145%. For comparison, the number of deaths caused by heart disease (the number one cause of death in the United States) has decreased by 9% in the same time period.^2^ AD is a chronic and irreversible neurodegenerative disorder with no current cure. The time delay between the start of the disease and the presentation of symptoms means that the disease is already at an advanced stage before it can be detected, and even when a patient presents with AD symptoms, a definitive AD diagnosis still remains challenging.

Postmortem diagnosis of AD is realized by the positive histological identification of both extracellular amyloid beta (Aβ) plaques and intracellular neurofibrillary tau tangles, which are both found in the brains of AD patients.^3^^,^^4^ A key step along the road to an early AD diagnosis would be an *in vivo* identification of Aβ plaques. However as the plaques are small (ranging from 10 to 200  μm)^5^ and located within the brain, this presents some logistical difficulties.

One recent idea to circumvent these difficulties is the use of the eye “as a window to the brain.” The retina and the brain are derived from the same embryological origin; they both extend from the neural tube. The retina is, therefore, the only part of the central nervous system that can be imaged noninvasively by optical methods. However, the question still remains whether the retina can hold the key to early AD diagnosis as there are few studies that directly correlate findings in the retina to those in the brain. Those studies that have been done before have found a correlation between the amplitude of retinal vascular pulsatility and neocortical Aβ scores (measured using florbetaben positron emission tomography),^6^ and also between fluorescent components (measured using fluorescence lifetime imaging ophthalmoscopy) and both p-tau181-protein concentration in the cerebral spine fluid and the mini-mental state examination score.^7^

Recent studies have also focused on the identification of extracellular Aβ accumulations in the retina of AD patients, however, there are conflicting reports on this topic.^8^ Some reports have identified extracellular Aβ in the retina^9^^–^^12^; however, other groups have demonstrated that there was no Aβ to be found.^13^^–^^16^ Another topic of current debate is whether AD could also be considered a vascular disorder,^17^ and therefore, the retinal vasculature of AD patients has also recently been studied. Patients with AD may exhibit more tortuous retinal vessels.^18^ Narrowing of retinal blood vessels and reduced venous blood flow rates have also both been found in AD patients,^19^^,^^20^ and an overall more sparse retinal microvascular network has been observed.^21^^,^^22^ A recent optical coherence tomography angiography (OCTA) study has shown a reduced vessel density specifically in the superficial capillary plexus.^23^ It has also been suggested that blood flow changes may precede neurodegeneration.^20^

Although the aforementioned studies have all demonstrated a reduction in blood circulation in the retina in late stage AD, recent studies have indicated that the retinal vessel density^24^ and vessel diameter^25^ both seem to be increased in individuals suffering from preclinical AD. Such results are consistent with the theory that an inflammatory response occurs in the retina in the early stages of AD—a theory that has also been proposed for neurovasculature.^26^^,^^27^

Retinal layer thinning, particularly in the retinal nerve fiber layer (RNFL), is also present in the retina of AD patients.^28^^,^^29^ However, looking forward to a marker for diagnosis, RNFL thinning is not specific to AD, and it is associated not only with other diseases, such as glaucoma^30^ and Parkinson’s disease,^31^ but also more generally with increasing age.^32^

With many contradictory observations, it is clear that there is still a great deal of research to be performed in order to fully understand the effects of AD on the retina. Some attempts to do this have focused on the use of mouse models of the disease. When performing studies on animal models, it is important to know where the similarities and differences to the human disease lie. Although there are many mouse models of AD,^33^ this work focuses on a doubly transgenic model that expresses a chimeric mouse/human amyloid precursor protein (APP) and a mutant human presenilin 1 (PS1).^34^^–^^36^ The following text therefore describes the retinal changes observed in this transgenic APP/PS1 mouse model so far.

Much like in the case of the human, while well documented in the brain,^37^^,^^38^ the appearance of extracellular deposits of Aβ in the retina is disputed. Several studies have reported no extracellular deposits of Aβ in the retina, despite plaques being present in the brain and an increased expression of APP in the retina, similar to what has been found in humans.^15^ This has been reported in mice of several ages: 9 months old,^39^ 7 to 12 months old,^40^ and 13 months old.^41^ It has been suggested that the nonamyloidogenic pathway may endogenously limit Aβ formation in the retina.^41^ A further extensive histological analysis of the retina concluded that no identifiable retinal pathology exists in these mice.^42^ Conversely, extracellular deposits of Aβ were found at the age of 27 months old in the choriocapillaris and the nerve fiber layer, but not in the other layers.^43^ In another study, plaques were found in the inner plexiform layer (IPL) and in the outer plexiform layer (OPL). These plaques ranged in size from 5 to 20  μm in mice of 12 to 13 months old, and larger with increasing age.^44^ This study reported no observed changes in retinal layer thickness. Deposits were also found distributed throughout the retina of transgenic mice at the age of 9 months and 17 months, but were identifiable as young as 2.5 months, even before the plaques appeared in the brain.^9^

There is, therefore, a need for more studies linking the retina and the brain in both AD patients and in animal models of AD,^45^ and more work needs to be done to assess and quantify the presence of Aβ in the retina.^46^ Optical coherence tomography (OCT)^47^ may be a useful tool to employ for this purpose. As a noncontact, noninvasive imaging modality, OCT has become part of clinical routine for *in vivo* retinal diagnostics. Functional extensions of OCT have made it possible to not only visualize contrasts based on backscattered intensity (reflectivity), but also motion (OCTA)^48^^–^^50^ and polarization properties [polarization-sensitive (PS-)OCT] such as birefringence.^51^^–^^54^ The birefringence of Aβ plaques has been studied in detail using polarimetry,^55^^–^^58^ and also with PS-OCT.^59^^,^^60^ The plaques appear as hyperscattering structures in standard reflectivity OCT images,^61^^–^^63^ but the addition of polarization-sensitive detection provides an additional tissue-specific contrast. Furthermore, the combination of PS-OCT with OCTA allows a simultaneous analysis of this tissue-specific contrast and changes in the retinal vasculature.

In this work, the appearance of the retina of an APP/PS1 mouse model of AD was evaluated by multicontrast spectral domain OCT. By observing retinal changes over a range of ages (45 to 104 weeks) in intensity-, motion- and polarization-based contrast modes, the observations in the retina were documented, mapped directly to histology, and compared to the Aβ plaque load in the brain.

## 2 Materials and Methods

### 2.1 Optical Coherence Tomography

A modified version of a PS-OCT system described elsewhere was used in this study.^64^ In brief, the system operated at a central wavelength of 840 nm with a full-width at half-maximum bandwidth of ∼100  nm, resulting in an axial resolution of around 3.8  μm in retinal tissue. Light incident upon the mouse eye was of a known polarization state, and the polarization-sensitive detection allowed for the differentiation between polarization-preserving tissue and polarization-altering tissue.

**Fig. 1:**
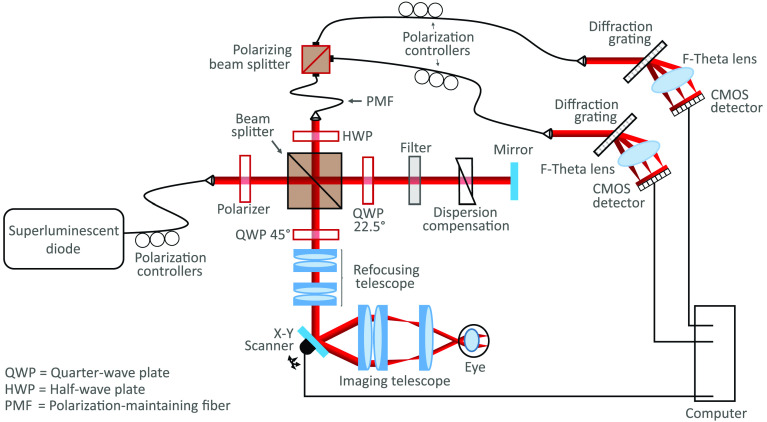
A modified version of the PS-OCT system first described by Fialová et al.^64^ A refocusing telescope was added to the system to allow focus correction of each individual mouse eye.

An additional refocusing telescope was added to the system to correct for myopia or hyperopia of the mouse eye.^65^ A diagram of the modified version of the system can be found in Fig. 1. Two additional achromatic doublet pairs (2× AC254-080-B, Thorlabs and 2× AC254-050-B, Thorlabs) were mounted on a translational stage, allowing the focus to be manually optimized for each individual mouse eye while reducing the uncorrected beam diameter incident on the pupil from 0.8 to 0.5 mm. The theoretical lateral resolution was, therefore, 5.6  μm for a mouse eye with no aberrations and a focal length of 2.6 mm.^66^ Refocusing with the additional telescope was theoretically most effective when correcting for −21 to −11 diopters or −3.8 to 9 diopters. Within these ranges, the beam diameter was kept between 0.25 and 1 mm, optimizing for both lateral resolution preservation and aberration minimization.^67^ Each mouse eye was aligned with respect to the 2.85-mW measurement beam to ensure the optic nerve head (ONH) was at the center of the 1  mm×1  mm field of view. With an A-scan rate of 83 kHz, five repeated B-scans (consisting of 512 A-scans each) were acquired at 400 unique locations. Such a scan pattern allowed for an increased signal-to-noise ratio (SNR) in the reflectivity and PS-OCT images and also the ability to produce OCTA images.

### 2.2 Mice

A breeding pair of APP/PS1 mice [(APPswe, PSEN1dE9), MMRRC stock number 34829-JAX] was purchased from the Jackson Laboratory (Bar Harbor, Maine),^34^^–^^36^ and a breeding colony was established at the Division of Biomedical Research at the Medical University of Vienna. Hemizygous mice were bred with wild-type siblings and subsequently kept under controlled lighting conditions (12-h light, 12-h dark) with food and water *ad libitum*. Both eyes of 24 mutant mice (17 females and 7 males) and 15 wild-type littermates (9 females and 6 males) were imaged using the multicontrast OCT system. At the time of imaging, the mice ranged in age from 45 weeks to 104 weeks. During the experiment, the animals were anesthetized using an inhalational isoflurane/oxygen mixture (4% isoflurane for 4 min in an induction chamber to induce anesthesia and 2% delivered via a nose cone thereafter). To facilitate the OCT imaging, pupils were dilated using topically applied tropicamide and phenylephrine. The cornea was kept moisturized using artificial tear eye drops, and heating pads were placed underneath the mice to prevent a reduction in body temperature. All experiments were performed in accordance with the ARVO Statement for the Use of Animals in Ophthalmic and Vision Research and Directive 2010/63/EU. Ethics protocols were approved by the ethics committee of the Medical University of Vienna and the Austrian Federal Ministry of Education, Science, and Research (BMBWF/66.009/0272-V/3b/2019).

### 2.3 OCT Image Analysis

**Fig. 2:**
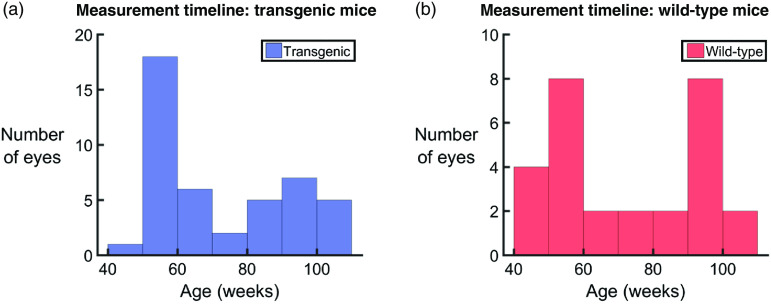
Histogram representations of the number of eyes used for analysis. (a) A total of 44 eyes from 24 APP/PS1 transgenic mice and (b) 28 eyes from 15 wild-type littermates were imaged within an age range of 45 to 104 weeks.

**Fig. 3:**
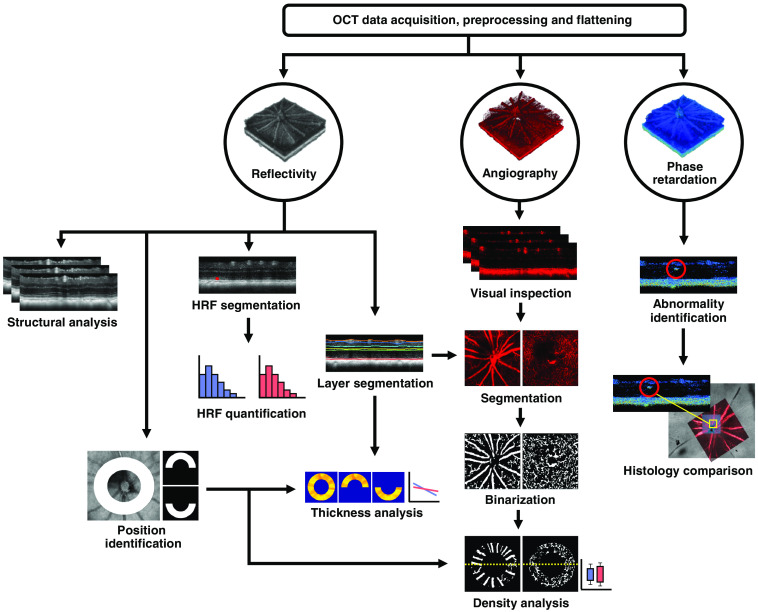
A flowchart of the multicontrast OCT postprocessing pipeline, which consists primarily of reflectivity, angiography, and phase retardation data. HRF, hyper-reflective foci.

The analysis performed in this work was based on a previously published multicontrast image processing pipeline including standard intensity-based reflectivity contrast, polarization-based contrast, and motion-based angiographic contrast.^68^ Prior to analysis, the images were corrected for axial motion and the retina was flattened with respect to the retinal pigment epithelium (RPE)/choroidal complex as detected by the cross-polarized channel,^69^ a technique made possible by the polarization sensitive detection. All preprocessing was performed on single images. Any dataset for which the retinal flattening failed (due to poor SNR) was excluded, resulting in a total of 72 datasets for evaluation (44 eyes from 24 transgenic mice and 28 eyes from 15 wild-type control mice). A graphical visualization of the age of the mice at each measurement can be found in Fig. 2. A flowchart of the overall postprocessing pipeline can be seen in Fig. 3, and a description of the analysis can be found in the following sections.

#### 2.3.1 Retinal thickness analysis

To allow for a comprehensive retinal thickness analysis, layer segmentation of the retina was first performed using a previously described algorithm.^69^ The distance between the inner limiting membrane and the posterior surface of the RPE was defined as the total retinal thickness. The posterior surface of the OPL served as the boundary between the inner and outer retina, as defined in this manuscript. An annulus around the ONH was created with an inner diameter of 500  μm and an outer diameter of 900  μm, and the mean total, inner, and outer retinal thicknesses were evaluated within this annular three-dimensional (3-D) volume. The annulus was then cut in half transversely and the mean thicknesses were calculated in the two resulting regions, corresponding to the superior and inferior retina. This boundary line is estimated to be accurate to within p/m 30 deg due to realignment to compensate for the orientation of the mouse eye. Coordinates of the segmentation lines and the ONH annuli were stored for later use in OCTA analysis.

To test for statistical significance, the retinal thickness measurements were plotted as a function of age for both the transgenic and the wild-type mice. A least-squares linear regression pretest was performed to determine if the thickness measurements were dependent on age. If the results were deemed significant (p<0.05), an analysis of covariance (ANCOVA) was performed to test for a difference in the trend between the two groups. In the case where the pretest was deemed statistically insignificant (p≥0.05), regular analysis of variance (ANOVA) was performed to test for a difference in the group means. The gradients of the slopes of the regression lines were also documented for both transgenic and wild-type mice, corresponding to a measurement of reduction of retinal thickness in units of μm per week.

#### 2.3.2 Hyper-reflective foci analysis

All 72 reflectivity datasets (44 eyes from 24 transgenic mice and 28 eyes from 15 wild-type control mice) were manually screened for hyper-reflective foci (HRF) in the posterior layers of the retina, spanning the region from the posterior OPL border to the posterior RPE (i.e., the whole outer retina). The HRF were then manually segmented using ITK Snap.^70^ Segmentation was performed on B-scans that were averaged 5 times at the same position. Using the data from this segmentation, the number and location of HRF was evaluated for each eye.

#### 2.3.3 OCT angiography

The B-scan repetition allowed for the computation of OCTA images, revealing locations of motion contrast. After bulk motion compensation and removal of frames with uncorrectable motion, OCTA images were computed by calculating the averaged magnitude of the complex differences between consecutive repeated B-scans. The time delay between the acquisition of repeated B-scans (≈7.7  ms from one B-scan to the next, including a scanner flyback time of 1.5 ms), which provides the angiographic contrast in the first place, also makes the method very susceptible to motion. All datasets were, therefore, manually visually screened, and data that included severe motion artefacts or regions of poor angiography signal were excluded. The angiography analysis was, therefore, performed on 39 transgenic eyes and 16 wild-type eyes.

An automated OCTA processing pipeline was created that consisted of several steps. The superficial vascular plexus (SVP) and the deep capillary plexus (DCP) were segmented from the retina using the layer segmentation coordinates obtained from the reflectivity data (SVP corresponds to the RNFL; DCP corresponds to the OPL). A maximum intensity *en face* projection was then calculated over the SVP and the DCP independently, and the histograms of each image were equalized using contrast limited adaptive histogram equalization^71^ before image binarization. The binary images were then morphologically opened and closed (disk-shaped structuring element with a radius of 1), and skeletonized to remove speckle noise and enhance the vessel connections. A square averaging filter with a 5-pixel side length was then applied to the images to create the final binary vessel representations of “vessel” versus “nonvessel.” The annuli around the ONH, which had been calculated using the reflectivity data, were then applied to these vessel maps. The vessel density was calculated as the percentage of pixels that were marked as “vessel” in the whole annulus, as well as in the superior and inferior retinal regions.

After this image processing was performed, a modified version of the Weber contrast^72^ was calculated to test the relationship of the OCTA vessel intensity to the background, using the binary image as a mask. The mean intensity value of all pixels determined both vessels ($\overset{¯}{I_{v}}$) and nonvessels ($\overset{¯}{I_{b}}$) was calculated, and the modified Weber contrast, $C_{W}$, was calculated as

$$C_{W}=\frac{\overset{¯}{I_{v}}-\overset{¯}{I_{b}}}{\overset{¯}{I_{b}}},$$ (1)

and the results were plotted as box-and-whisker diagrams for both transgenic and wild-type mice. The contrast measurement was performed to ensure no systematic errors were present that would result in different angiogram intensities between transgenic and wild-type groups.

#### 2.3.4 Polarization properties

Images depicting the phase retardation, *δ*, were calculated for every B-scan by

$$\delta =\arctan(\frac{A_{V}}{A_{H}}),$$ (2)

where $A_{V}$ and $A_{H}$ correspond to the signal amplitudes of the co- and cross-polarized channels, respectively.^51^^,^^73^ In the healthy mouse retina, high retardation values are only expected in the melanin-containing regions, namely the RPE, the choroid, and the remnants of the hyaloid artery near the ONH. Depolarization occurs due to the fact that the melanin granules scramble the polarization state of the incoming light beam, resulting in random phase retardation values. Polarization-preserving tissues, i.e., the rest of the retina, do not retard the phase of the incident beam, and therefore, the phase retardation values are low. All retardation B-scans were manually inspected for abnormally high retardation signals from outwith these retinal layers. The number of these abnormally high retardation signals was evaluated, and the sources were investigated with retinal histology.

### 2.4 Histology and Immunostaining

After OCT imaging, a subgroup of the mice (14 transgenic, age: 54 to 104 weeks, 7 wild-type control, age: 54 to 103 weeks) were euthanized by cervical dislocation. Immediately after sacrifice, the brains were extracted, and the eyes were enucleated for histological analysis.

#### 2.4.1 Brain

The mouse brains were sagittally cut into two hemispheres, and one hemisphere was prepared for histopathological workup. The samples were fixed in 4% formalin and processed through graded alcohols and xylene into paraffin. Sagittal brain sections with a thickness of 2.5  μm were cut on a microtome, deparaffinized, rehydrated, and stained immunohistochemically using an anti-Aβ antibody (clone 6F/3D, diluted 1:100, Dako). The sections were evaluated using a slide scanner (Hamamatsu NanoZoomer 2.0 HT) and saved for digital pathology. The images were analyzed using Fiji.^74^ First, the cortex was manually selected and the “ColSeg” tool^75^ was utilized to segment the plaques by their brown color. The “analyze particle” tool was then used to count the plaque number and calculate the plaque load in plaques per ${\mathrm{mm}}^{2}$. The plaque load was then plotted as a function of mouse age, and a linear regression analysis was performed on the data.

#### 2.4.2 Retina

To obtain vertical histological slices of the retina, six left eyes from wild-type (n=3) and transgenic (n=3) mice diagnosed with and without abnormal OPL banding (see Sec. 3.5) were immersed unopened in Davidson’s fixative for 24 h at 4°C and processed through graded alcohols and xylene into paraffin. Three-micron-thick sections were then cut, mounted onto slides, deparaffinized, rehydrated, and stained with hematoxylin and eosin (H&E).

For wholemount preparation and Aβ immunostaining, eyes were immersed in 4% paraformaldehyde (PFA) in 0.1 M phosphate buffered saline (PBS), pH 7.4. Some eyes were fixed unopened. In the others, the cornea and lens were removed and the eyecups were fixed in PFA for at least 24 h at room temperature. After rinses in PBS, the retina was dissected free from RPE, choroid, and sclera, cryoprotected in ascending sucrose concentrations (10%, 20%, and 30%), and snap-frozen and thawed 3 times to increase antibody penetration. For each mouse, the left retina was treated with 70% formic acid for 10 min and then rinsed repeatedly in PBS, while the right retina was left untreated. Retinal wholemounts were processed free-floating in 24-well plates and all incubations and rinses were done with gentle rotation on a rocker table at 4°C. Blocking of nonspecific binding was performed in 3% normal donkey serum in 0.1 M PBS, 0.25% Triton X-100, and 0.05% sodium azide (medium), followed by incubation with mouse anti-human Aβ (Abcam, ab11132, clone DE2B4, 1:400 in medium) for 72 h. After washes in PBS, retinas were incubated in donkey anti-mouse Fab fragments conjugated with Alexa Fluor 488 (Jackson ImmunoResearch Laboratories, 1:500 in medium) for 24 h, rinsed, and coverslipped (retinas ganglion cell side up) in Aqua/Polymount (Polysciences). To serve as positive and negative controls, respectively, brains from transgenic APP/PS1 mice and their wild-type littermates were harvested after enucleation and fixed in 4% PFA for 24 h at 4°C. After washes in PBS, brains were cryoprotected in ascending sucrose concentrations (10%, 20%, and 30%), snap-frozen in liquid nitrogen-prechilled isopentane, and cut into 100-μm-thick sections using a cryotome. The sections were collected in PBS/0.05% sodium azide and processed under the same conditions applied to retinal wholemounts. H&E-stained sections were examined with brightfield illumination on a Zeiss Axio Imager Z2. Immunofluorescence analysis was performed with a Zeiss LSM880 laser scanning microscope (LSM). A total of 17 retinas from 11 transgenic mice (age: 54 to 103 weeks) and 11 retinas from 6 wild-type control mice (age: 54 to 103 weeks) were suitable for detailed histological examination. A 1:1 correlation of the retinal wholemounts to the OCT image data was then performed by mapping the vessel pattern of the SVP as visualized by the LSM to the corresponding OCTA datasets.

## 3 Results

### 3.1 Retinal Thickness

**Fig. 4:**
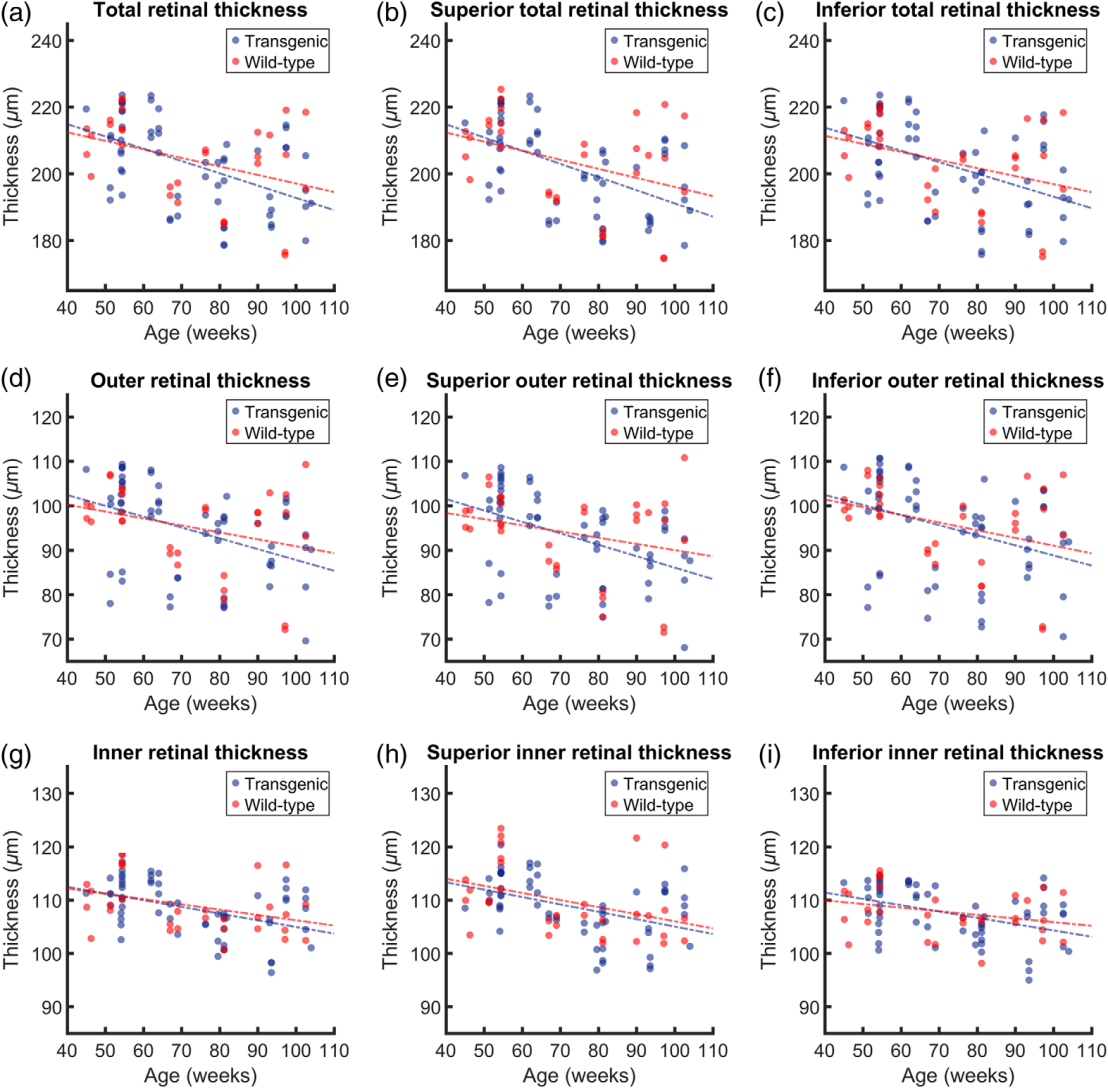
Analysis of retinal thickness as a function of age for both transgenic and wild-type mice. Total retinal thickness measured around (a) the whole annulus, then subdivided into (b) a superior half and (c) an inferior half. Outer retinal thickness measured (d) around the whole annulus, (e) in the superior half and (f) in the inferior half. Inner retinal thickness measured (g) around the whole annulus, (h) in the superior half and (i) in the inferior half. The corresponding statistical evaluation can be found in Tables 1 and 2, and the gradients of the slopes can be found in Table 3.

The total, outer, and inner retinal thicknesses were calculated in an annulus around the ONH, and also for the superior and inferior (180±30) deg sectors, resulting in nine thickness comparisons between transgenic and wild-type mice. The results of this analysis can be found in Fig. 4. When plotted as a function of age, all nine datasets displayed a general trend of decreasing retinal thickness with age for both the transgenic mice and the wild-type controls. Statistical pretests revealed that the dependence upon age was significant for all data except three wild-type datasets: the total outer retinal thickness (p=0.075), the superior outer retinal thickness (p=0.124), and the inferior inner retinal thickness (p=0.101). For these three datasets, the comparison between transgenic and wild-type data was analyzed with ANOVA, whereas all other analysis was performed with ANCOVA. No statistical significance was found between the retinal thickness changes in the transgenic and control groups, for any of the retinal regions. The results of the statistical pretests can be found in Table 1, and the comparisons can be found in Table 2. Using the gradient of the slope of the linear regression analysis, a measurement of the decrease of retinal thickness was documented in units of μm per week, as shown in Table 3. All trends were negative; therefore, the gradient of each slope is the negative of the value in this table. Despite a general trend of faster retinal thinning in the transgenic groups (Table 3), the results outlined in Table 2 show that this is not statistically significant.

**Table 1:** Statistical pretests for the retinal thickness (RT) analysis. All values are p-values which were calculated using linear regression analysis. Significance was defined as p<0.05.

		Position					
		Total		Superior		Inferior	
		Transgenic	Wild-type	Transgenic	Wild-type	Transgenic	Wild-type
Retinal thickness	Total	$6.146\times 10^{-5}$	0.033	$1.598\times 10^{-5}$	0.037	$2.858\times 10^{-4}$	0.034
	Outer	$9.010\times 10^{-4}$	0.075	$2.159\times 10^{-4}$	0.124	0.004	0.045
	Inner	$3.998\times 10^{-4}$	0.038	$5.437\times 10^{-4}$	0.030	$7.544\times 10^{-4}$	0.101

**Table 2:** p-values comparing retinal thickness between transgenic and wild-type groups. No statistically significant changes in retinal thickness were found between groups.

		Position		
		Total	Superior	Inferior
Retinal thickness	Total	0.421^a^	0.392^a^	0.466^a^
	Outer	0.727^b^	0.716^b^	0.647^a^
	Inner	0.640^a^	0.936^a^	0.835^b^

a p-values from ANCOVA comparing the trends of the transgenic retinal thickness versus age to the wild-type retinal thickness versus age.

b p-values from one-way ANOVA due to the failure of the statistical pretests. Significance was tested for between the means of the two groups.

**Table 3:** Decrease in retinal thickness in units of μm per week.

		Position					
		Total		Superior		Inferior	
		Transgenic	Wild-type	Transgenic	Wild-type	Transgenic	Wild-type
Retinal thickness	Total	0.37	0.26	0.40	0.27	0.35	0.24
	Outer	0.24	0.16	0.26	0.14	0.23	0.17
	Inner	0.13	0.10	0.14	0.13	0.12	0.07

### 3.2 Hyper-Reflective Foci

**Fig. 5:**
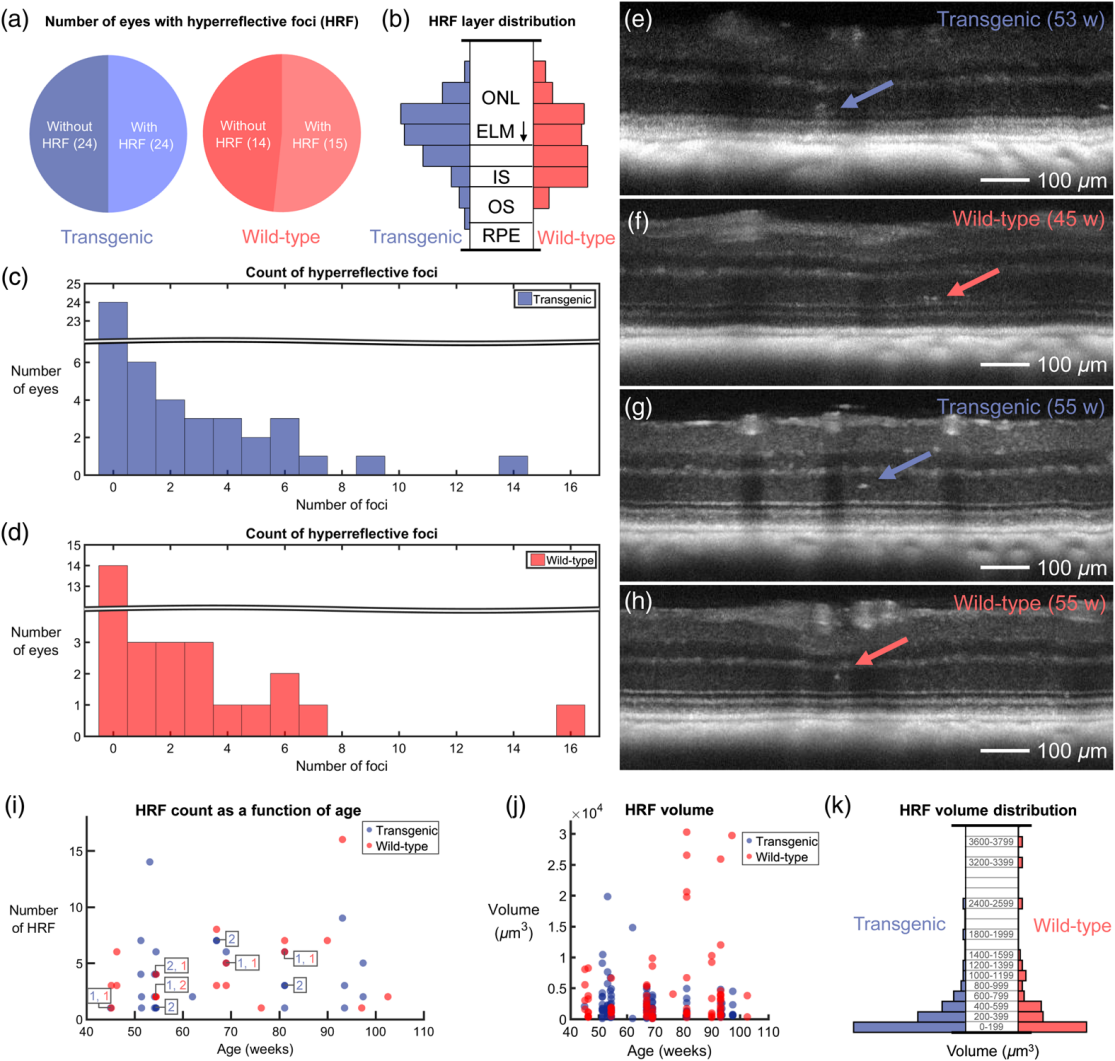
Results of HRF analysis. (a) Pie charts indicating the number of eyes with and without HRF for both transgenic and wild-type mice. (b) HRF probability distribution displayed with respect to outer retinal layer position for transgenic and wild-type mice. The distributions are very similar, with most HRF appearing near the ELM. ONL, outer nuclear layer; IS, inner segments; OS, outer segments; and RPE, retinal pigment epithelium. (c) Histogram of HRF occurrence in transgenic mice. (d) Histogram of HRF occurrence in wild-type mice. (e)–(h) Some examples of the appearance of HRF in OCT reflectivity images. Each image is a maximum intensity projection over four consecutive B-scans, where each B-scan is already averaged 5 times and plotted on a logarithmic scale. HRF located above the ELM in (e) both the transgenic mouse retina and (f) the wild-type retina. HRF located in the middle of the ONL in both (g) the transgenic mouse retina and (h) the wild-type retina. Ages of mice in weeks (w) are indicated in (e)–(h). (i) HRF count as a function of age. Overlapping datapoints are indicated with color-coded numbers. (j) HRF volume plotted as a function of mouse age. (k) HRF volume distribution for transgenic and wild-type mice. In (j) and (k), one data outlier was excluded (wild-type, age: 81 weeks, HRF volume: $7.3\times 10^{4}\;\mu m^{3}$).

For each retina, the whole $1\times 1\;{\mathrm{mm}}^{2}$ area surrounding the ONH was evaluated. Of the 24 mutant mice, 16 showed HRF in at least one eye. In the wild-type littermate control group, HRF were identified in 12 out of the 15 mice. Figure 5(a) displays pie charts that document this in terms of eyes; there were an equal number of eyes with and without HRF in the transgenic mice, and a difference of only one in the wild-type mice. Since it is difficult to identify small HRF in the plexiform layers due to the appearance of hyper-reflective blood vessels, a normalized probability distribution of all identified HRF in the outer retina alone was plotted [Fig. 5(b)]. A similar HRF distribution in transgenic and wild-type retinas was observed. The number of HRF per eye was also counted for the transgenic [Fig. 5(c)] and the wild-type [Fig. 5(d)] mice. With the exception of one outlier in each group, all outer retinas contained <10 HRF within the investigated field of view. Qualitatively, the types of HRF also looked very similar between transgenic and wild-type mice, examples of which can be found in Figs. 5(e)–5(h). In these images, maximum intensity projections over 4 consecutive B-scans are displayed to remove speckle noise. Figures 5(e) and 5(f) show examples of larger HRF located anterior to the external limiting membrane (ELM) in the transgenic and wild-type animals, respectively, whereas Figs. 5(g) and 5(h) show smaller HRF in the middle of the ONL. Neither the number of HRF nor their volume correlated with the age of the mice, for either group [data shown in Figs. 5(i)–5(j)]. Histograms documenting HRF volume for transgenic and wild-type groups can be found in Fig. 5(k); both groups display a similar volume distribution.

### 3.3 OCT Angiography

**Fig. 6:**
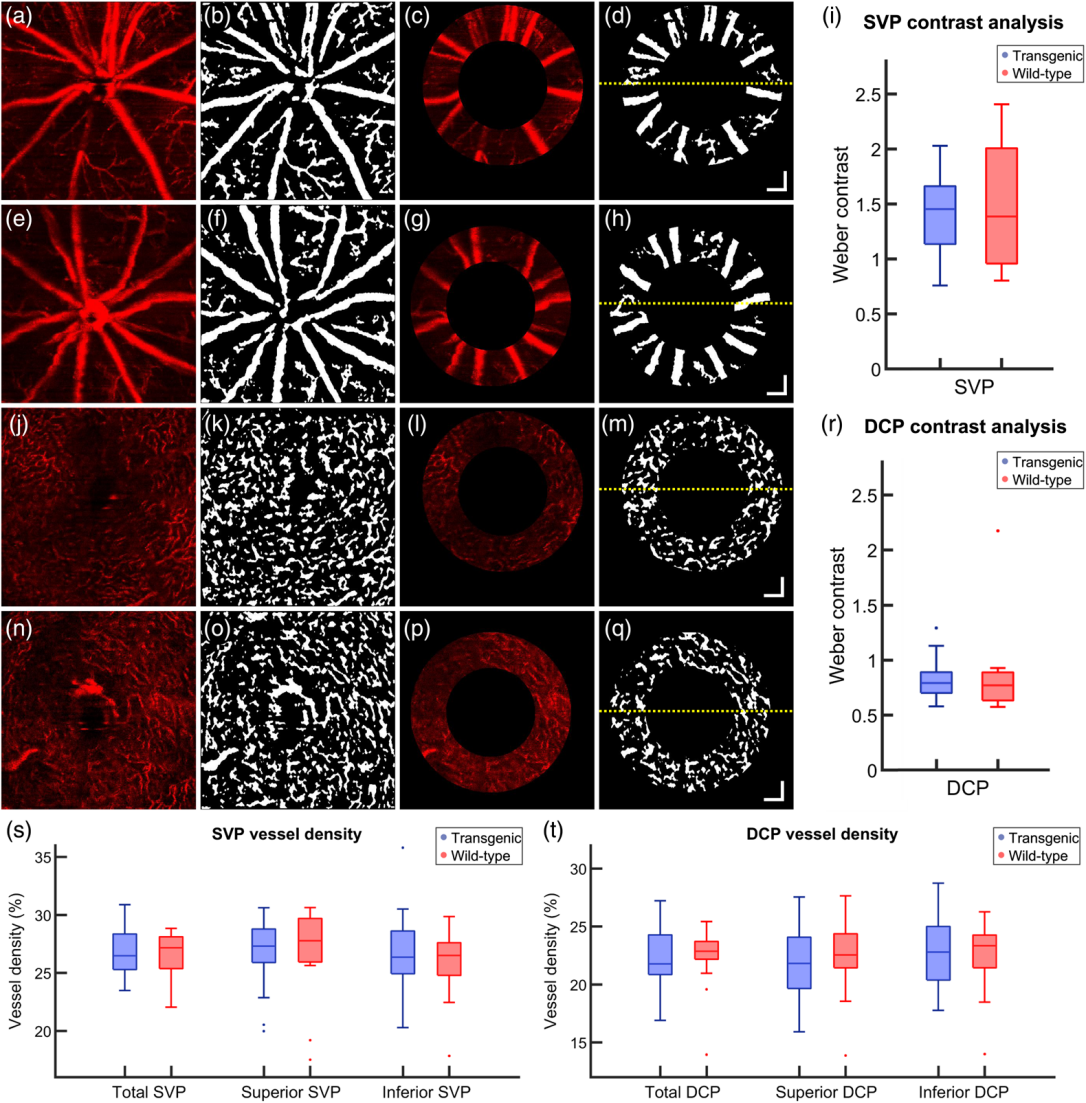
(a)–(h) Example of OCTA analysis of the SVP of a (a)–(d) transgenic mouse and (e)–(h) a wild-type control. (a), (e) *En face* OCTA depth projection through the SVP. (b), (f) Binary representation of the SVP with white pixels corresponding to blood vessels. (c), (g) Annulus around the ONH as provided by the intensity-based contrast data. (d), (h) Binarized annulus, where the yellow dashed line corresponds to the boundary between the superior retina (above) and the inferior retina (below). (i) Weber contrast comparing the intensity of the angiogram signal of the blood vessels to the intensity of the background in the SVP. (j)–(q) Example OCTA analysis of the DCP of a (j)–(m) transgenic mouse and a (n)–(q) wild-type control. (j), (n) *En face* OCTA depth projection through the DCP. (k), (o) Binary representation of the DCP with white pixels corresponding to blood vessels. (l), (p) Annulus around the ONH as provided by the intensity-based contrast data. (m), (q) Binarized annulus, where the yellow dashed line corresponds to the boundary between the superior retina (above) and the inferior retina (below). (r) Weber contrast comparing the intensity of the angiogram signal of the blood vessels to the intensity of the background in the DCP. (s)–(t) Vessel density analysis. Total, superior, and inferior vessel density calculated for transgenic and wild-type mice in the (s) SVP and the (t) DCP. Age (a)–(d), (j)–(m): 93 weeks, age (e)–(h), (n)–(q): 76 weeks. Single points in (r)–(t) correspond to data outliers. All scale bars=100  μm.

Following layer segmentation of the SVP and the DCP, the vessel density was quantified in these layers in the total, superior, and inferior retina for both transgenic and wild-type mice. Figure 6(a) shows a typical OCT angiogram from the SVP of a transgenic mouse. Combining its binarized form [Fig. 6(b)] with the annulus, which was defined from the reflectivity data [Fig. 6(c)], binary representations of the retinal vasculature around the ONH were obtained [Fig. 6(d)]. Figures 6(e)–6(h) show the same processing steps for a wild-type mouse. The binarized annulus [Figs. 6(d) and 6(h)] was then used as a mask on the annular angiogram [Figs. 6(c) and 6(g)] in order to calculate the Weber contrast [Fig. 6(i)]. The contrast is similar between transgenic and wild-type mice. Figures 6(j)–6(m) and 6(n)–6(q) show examples of the same analysis pattern for the DCP in the transgenic and wild-type mice, respectively. The Weber contrast in this case [Fig. 6(r)] is lower for both groups, although the contrast values remain similar between transgenic and wild-type mice.

The vessel density calculations for all retinas in the SVP and the DVP are shown in Figs. 6(s) and 6(t), respectively. No significant differences were observed between transgenic and wild-type mice for any of the retinal regions.

### 3.4 Phase Retardation Abnormalities

**Fig. 7:**
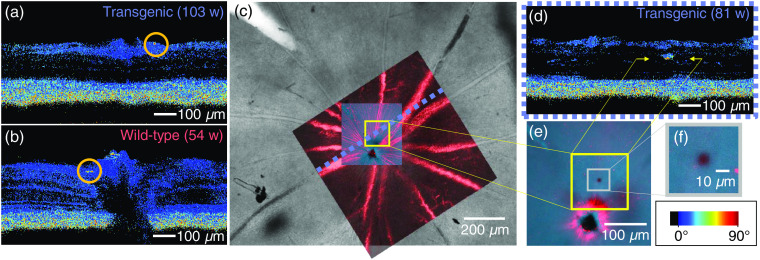
Depolarizing deposits. (a) Example of depolarization along a vessel wall (indicated by orange circle). (b) Example of depolarization near the ONH (indicated by orange circle). (c) Identification of migrated melanin. After wholemounting the retina, the OCT angiography data (in red) were used to correlate the vessels measured *in vivo* to the overview of the retina provided by the *ex vivo* preparation (gray scale). (d) PS-OCT image showed a location of abnormally high-phase retardation in the INL (indicated by yellow arrows). Scale bar in bottom right applies to (a), (b), and (d). (e) A high-resolution confocal microscopy scan was acquired at the area of interest marked in (c), at the depth position marked by the yellow arrows in (d). A cluster of melanin is revealed at this location, as seen in (f).

All individual B-scans of all mouse retinas were screened for phase retardation abnormalities, i.e., depolarizing deposits located outwith the known melanin-containing RPE and ONH regions. Such deposits were found in at least one eye of 22 out of 24 transgenic mice and 11 out of 15 controls, and there were no apparent differences between those deposits observed in transgenic and wild-type groups. Figures 7(a) and 7(b) show the two most common forms of phase retardation abnormalities, which appear as small, round depolarizing deposits beside a vessel wall [Fig. 7(a)] or beneath the RNFL adjacent to the ONH [Fig. 7(b)]. No Aβ plaques were identified in any of the retinas where the PS-OCT data were correlated to histology (more details in Sec. 3.6). However in some cases, melanin migration was found to be the source of the contrast, as demonstrated in Figs. 7(c)–7(f).

### 3.5 Double-Banded OPL

**Fig. 8:**
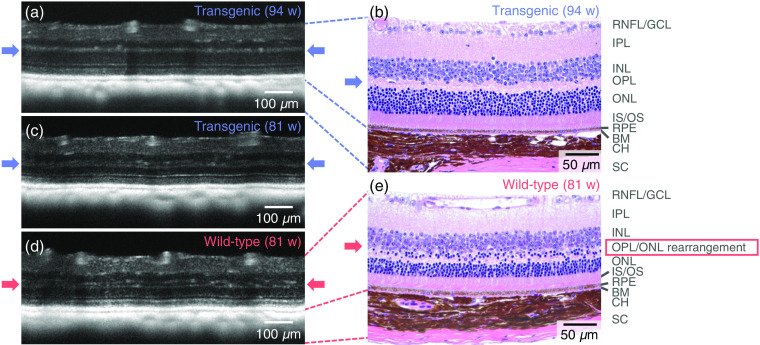
Demonstration of retinal layer abnormalities. The OPL is indicated with arrows. (a) A transgenic mouse retina with a typical appearance—the outer plexiform layer appears as one single hyper-reflective band. (b) H&E-stained histological slice of the same mouse retina as in (a). (c) A transgenic mouse retina with the OPL disrupted, appearing as a double-banded hyper-reflective layer. This effect was observed in 3/24 transgenic mice. (d) A similar double-banding effect was also observed in 3/15 wild-type littermates. (e) H&E-stained histological slice of the same mouse retina as in (d). The structural correlate of the double-banded OCT signal in the OPL region appears to be rearranged proximal outer nuclear layer somata. RNFL, retinal nerve fiber layer; GCL, ganglion cell layer; IPL, inner plexiform layer; INL, inner nuclear layer; OPL, outer plexiform layer; ONL, outer nuclear layer; IS/OS, inner/outer segment junction; RPE, retinal pigment epithelium; BM, Bruch’s membrane; CH, choroid; and SC, sclera. Age: (a)–(b) 94 weeks and (c)–(e) 81 weeks.

Abnormalities in the structure of the ONL/OPL were found in the reflectivity OCT images in both eyes in a total of 3/24 transgenic mice (age: 54 weeks, 67 weeks, and 81 weeks) and 3/15 wild-type control (age: 67 weeks, 81 weeks, and 97 weeks). Examples of the appearance of the double-banded OPL can be found in Fig. 8. Figure 8(a) shows an example of a “normal” appearance of a retina observed in a transgenic mouse, where the OPL appears as a single hyper-reflective band. After H&E staining, it was confirmed that the retinal layer structure appeared as expected [Fig. 8(b)]. In contrast, the hyper-reflective OPL appears to split into two in Fig. 8(c). Similar double bands of hyper-reflective OPL signal were observed in the three wild-type mice as shown in Fig. 8(d). To evaluate potential structural bases underlying the atypical retinal layer contrast, mouse retinas depicting OPL double-banding in the OCT exam were also embedded in paraffin, sectioned, and stained with H&E. Microscopical examination revealed that the double-banding of the OPL precisely correlated with a rearrangement of proximal ONL somata toward the outer border of the inner nuclear layer (INL) [Fig. 8(e)].

### 3.6 Retinal Histology

#### 3.6.1 Typical observations

To confirm human Aβ in the retinas of APP/PS1 transgenic mice, indirect immunofluorescent staining of retinal wholemounts was performed with a mouse monoclonal antibody directed against amino acids 1 to 17 of human Aβ (clone DE2B4). The marker identifies intracellular $A\beta^{83}$ and safely detects extracellular Aβ without cross-reacting with ${\mathrm{APP}}^{83}$. This is evidenced by the clear labeling of Aβ plaques only in brain sections from APP/PS1 transgenic animals used as a positive control.

**Fig. 9:**
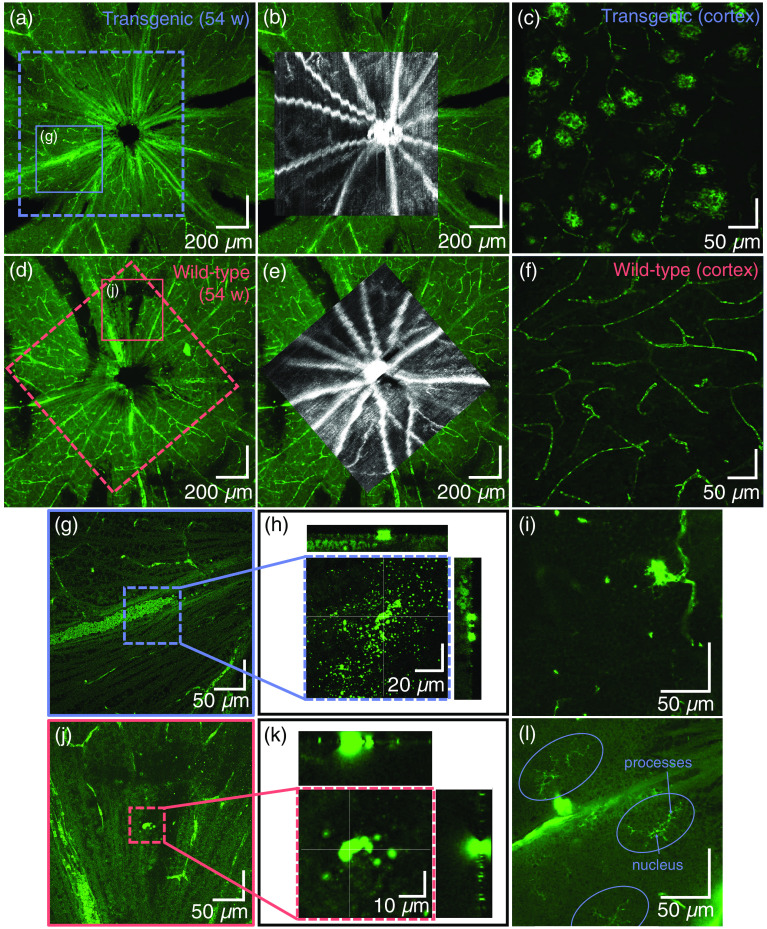
Representative depictions of the *ex vivo* retina following fluorescent staining against Aβ. (a) Retinal wholemount of a transgenic mouse and (b) its correlation to *in vivo* OCT data. (c) The positive control (the cortex of the transgenic mouse) shows fluorescent labeling of Aβ plaques and capillaries. (d) Retinal wholemount of a wild-type mouse and (e) its correlation to *in vivo* OCT data. (f) In the cortex of the wild-type mouse (negative control), only capillaries are labeled. (g)–(l) Some typical observations seen throughout transgenic and wild-type mouse retinas. (g) When zooming in to the surface of the retina in the location indicated in the dashed box (h), some scattered bright spots appear. The orthogonal views (positions indicated by the white cross hairs), however, show that these lie only on the surface of the retina. (i) Fluorescent signal positioned at a capillary junction. (j)–(k) Similar to (g)–(h), single, larger accumulations of fluorescent tracer find themselves at the interface of vitreous and retina, but not within the retina. (l) Microglia, indicated by ovals, are identifiable by their dendritic processes and are also found throughout the retina. This image was acquired within the GCL.

Following the donkey anti-mouse secondary antibody staining protocol outlined in Sec. 2.4.2, it was expected that in addition to Aβ, sources of endogenous IgG present in the retina would bind to the secondary anti-mouse antibody and be highlighted. Figure 9(a) shows an example of a retinal wholemount of a transgenic mouse with the peripapillary blood vessels distinctly labeled due to abundant endogenous mouse IgG present in the serum. Figure 9(b) demonstrates the precise fit of the *ex vivo* vessel pattern with the *in vivo* OCTA image. The wavy appearance of some of the vessels is a result of motion artefacts caused by breathing during the measurement. From the positive control of the cortex of the transgenic mouse [Fig. 9(c)], it can already be observed that signal of a similar intensity to the roundish Aβ plaques also comes from the capillary network. Figures 9(d)–9(f) shows the equivalent images for an example of a wild-type control mouse. In the cortex of the wild-type mice [used as a negative control, Fig. 9(f)], only the capillary network showed fluorescent labeling.

Outwith the blood vessels and capillary network, other sources of fluorescent signal were found in both transgenic and control mice. Examples of such features are shown in [Figs. 9(g)–9(l)]. In Fig. 9(a), a blood vessel (indicated by the solid box) appears to be intensely fluorescing. However, by analyzing a series of confocal optical sections (z-stacks) throughout this region [Figs. 9(g) and 9(h)], it became clear that this signal derived from aggregates of secondary antibody–fluorochrome conjugate artefactually adhering to the surface of the retinal GCL. The signal did not derive from either neuronal or non-neuronal structures within the retina as the overview at 10× magnification made it appear. Similar observations were made in the wild-type retinas too [Figs. 9(j) and 9(k)]. Structures signaling intensely from within the retina included areas where branches of retinal capillaries appeared to get close together, reminiscent of microaneurysms [Fig. 9(i)], and microglia [Fig. 9(l)], identifiable by the dendritic morphology of their processes. Any source of fluorescent signal which did not fall under one of these categories was then considered a candidate for Aβ.

#### 3.6.2 Potential deposits of retinal amyloid beta

**Fig. 10:**
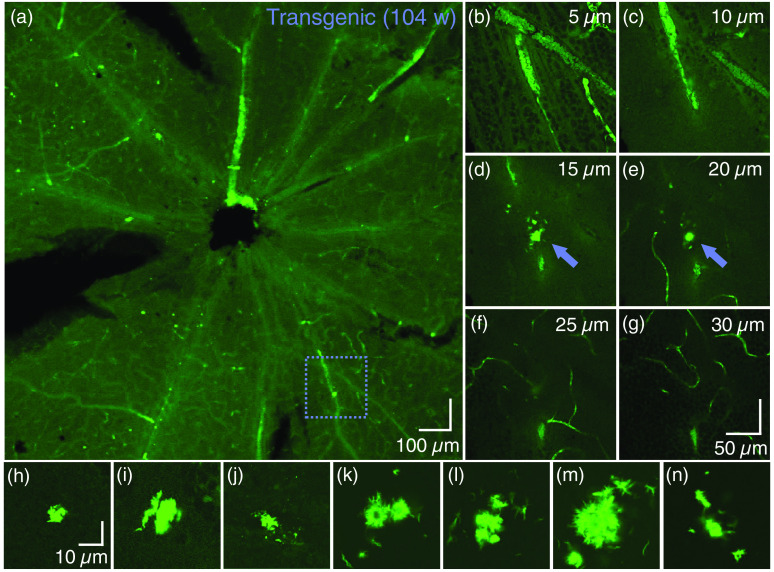
Candidates for fibrillary Aβ detected in the retina of one mouse, as identified by confocal microscopy. (a) Overview of the *ex vivo* retina (left eye) acquired with a 10× magnification objective lens. (b)–(g) *En face* planes at 5  μm intervals at the position identified by the dashed box in (a), where the zero-position is at the interface of vitreous and GCL. The fluorescent abnormality, i.e., the Aβ candidate, is indicated by the arrow in (d) and (e). Scale bar in (g) is valid for (b)–(g). Images were acquired with a 40× magnification objective lens. (h)–(n) Seven further Aβ candidates were identified in the retina of the right eye of the same mouse (all acquired with a 40× magnification objective lens). All structures were detected <40  μm from the surface of the retina, i.e., between the RNFL and IPL. Scale bar in (h) is valid for (h)–(n).

Of the 17 mice which underwent retinal histology, only one mouse (transgenic, age: 104 weeks) displayed fluorescent signals in the retina which could be attributed to extracellular Aβ. In this mouse, there was one such area of interest in the left eye, and seven in the right eye. Images of all Aβ plaque candidates can be found in Fig. 10. Figure 10(a) shows an overview of the left retina. Although many bright spots were observed, only the one indicated by the dashed box did not exhibit the characteristics of what was shown in Fig. 9. A z-stack through the retina confirmed that this plaque candidate sat ∼15  μm below the surface of the retina, extending into the anterior IPL. Examples of z-planes can be found in Figs. 10(b)–10(g). A similar analysis of the right retina provided images of the further seven plaque candidates, which can be seen in Figs. 10(h)–10(n). The locations of three of the eight plaque candidates were also covered in the field of view of the *in vivo* OCT measurements, however, no abnormalities were found in these locations in the OCT data, using any mode of contrast.

### 3.7 Cortical Amyloid Beta Plaque Load

**Fig. 11:**
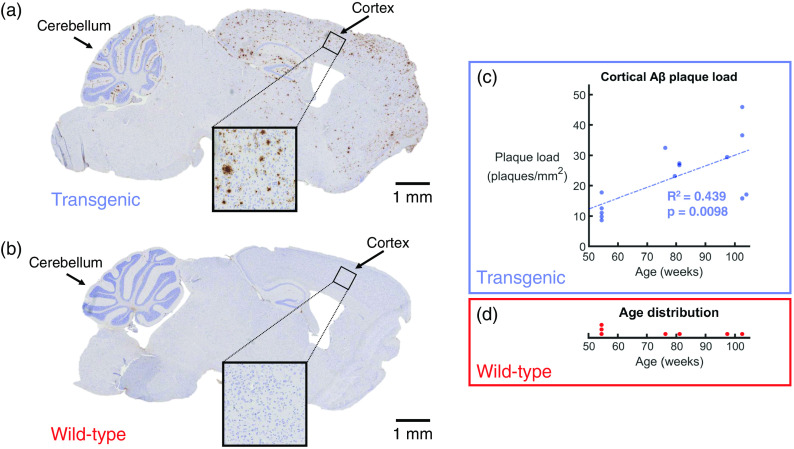
Quantification of the plaques per ${\mathrm{mm}}^{2}$ in the cortex. (a) Histological slice of a transgenic mouse brain, immunohistochemically stained against Aβ. Aβ plaques appear as brown deposits. Age: 103 weeks. (b) Following the same staining protocol, the wild-type littermates do not show any Aβ plaques in the cortex. Age: 103 weeks. (c) Count of plaques per ${\mathrm{mm}}^{2}$ for a subset of 14 transgenic mice. Linear regression analysis showed a statistically significant trend of an increasing plaque load with age ($R^{2}=0.439$, p=0.0098). (d) The age distribution of the seven wild-type control mice that were examined, none of which showed any Aβ plaques.

In a subset of the mice (14 transgenic mice and 7 wild-type littermates), histological slices of the brain were prepared and immunohistochemically stained against Aβ. Figure 11(a) shows an example of the staining results for a transgenic mouse, and Fig. 11(b) shows an example of a similar region in the control brain. In the transgenic mice, brown plaques were identifiable throughout the entire cortex. The mouse depicted in Fig. 11(a) was 103 weeks old at the time of sacrifice, and Aβ plaques were also visible in abundance in the hippocampal formation and the cerebellum, as well as in other areas of the brain. To the contrary, no plaques were observed in any of the brain regions in the seven examined wild-type mice [Fig. 11(b)].

For the 14 transgenic mice, the plaque load was plotted as a function of age. This plot can be found in Fig. 11(c). Linear regression analysis revealed an $R^{2}$ value of 0.439 and a p value for the significance of the gradient of the slope of 0.0098. This model indicated that the plaque load increases by 0.354 plaques per ${\mathrm{mm}}^{2}$ per week over the investigated age range. Such a result demonstrates that Aβ plaque load increases with age in the transgenic mouse brain, and that the trend is statistically significant. The age distribution of the seven wild-type mice can be found in Fig. 11(d).

## 4 Discussion

Since the role of the retina in Alzheimer’s disease is still widely disputed, the aim of this work was to provide a comprehensive overview of what can be observed in the retina of an APP/PS1 mouse model using multicontrast OCT and to compare this to histological results. A combination of reflectivity images, PS-OCT images, and OCTA was used to investigate the structure and function of the retina. From the *in vivo* data alone, several retinal abnormalities were successfully identified in this model, however, there were no statistically significant differences between the transgenic and wild-type groups. This suggests that the HRF, retinal thickness changes, phase retardation abnormalities, and structural differences which were measured with OCT are either strain-related or age-related, rather than being due to the genetic mutation itself.

The total retinal thickness was found to significantly decrease with age in the superior and inferior halves as well as in the whole annulus around the ONH. No difference was seen, however, between the transgenic and wild-type groups. These results provide an *in vivo* validation for that which was previously observed *ex vivo* by Perez et al.^44^ In future studies, the inner and outer retinas could be subdivided further to quantify individual layer thickness. Since RNFL thinning occurs in AD patients,^28^^,^^29^ it may also be interesting to quantify the RNFL thickness alone in this mouse model. However, quantifying the RNFL thickness in mice using OCT is difficult, as the peripapillary thickness of the healthy RNFL is only ∼20  μm^76^ and is interrupted by blood vessels and ganglion cells. Automatic segmentation of the mouse RNFL is, therefore, challenging, as it cannot always be distinguished from the IPL in the OCT images. Owing to its fibrous structure, the RNFL is also birefringent and since it is much thicker in the human retina, the effect of the birefringence is stronger. PS-OCT has already been proposed as a tool for imaging the RNFL in glaucoma,^77^^,^^78^ so RNFL measurements in AD patients may be a promising future application for PS-OCT.

The phase retardation analysis performed in this study mainly identified depolarization deposits associated with vessel walls and melanin pigments. No depolarizing deposits were identified in locations which corresponded to candidates for retinal Aβ, although locations of melanin migration which were observed in this study mirror results which were documented in the human in age-related macular degeneration.^79^ Future experiments on this topic could also consider HRF in the intensity data, but this becomes challenging in layers which contain vasculature, as the edges of vessels can also appear as hyper-scattering features. Previous PS-OCT studies identifying migration of melanin pigments have typically used the metric of degree-of-polarization-uniformity (DOPU) to quantify the polarization scrambling, or depolarization, of the structure in question.^68^^,^^79^^,^^80^ In order to calculate DOPU, a sliding window must be applied within the images, sacrificing the overall resolution. In this study, as the depolarizing structures were small, it was decided to use the phase retardation images alone, as they provided higher image resolution.

The OCTA analysis revealed no significant differences in the vessel density between transgenic and wild-type groups in either the SVP or the DCP. This is contrary to what has been observed in human vessel microangiography studies.^22^^,^^23^ However, in the previous human studies, the area of interest usually targets the fovea. Since mice do not have a fovea, there is not an equivalent measurement in the mouse retina. Although our vessel density measurements fall within the range of those which have previously been reported for wild-type mice,^81^ there has not yet been a detailed analysis of the expected vessel densities for the SVP and DCP in old APP/PS1 mice. We, therefore, used the modified Weber contrast metric to ensure that our similar densities between transgenic and wild-type retinal vasculature were not due to external factors, such as cataract formation. The modified Weber contrast was similar between transgenic and control groups; the wider standard deviation for the wild-type can be attributed to the smaller sample size. The Weber contrast in the DCP was lower than in the SVP for both groups, which is to be expected as the blood flow in the DCP is lower than in the SVP, and therefore, the angiogram signal is weaker due to the more random orientation of the red blood cells. It would be of interest to perform measurements of blood flow in this mouse model, for example, using a Doppler OCT system, as has been performed in the retina of human AD patients.^19^^,^^20^ Measurements of vessel tortuosity^18^ would also be an interesting future direction of research with this mouse model. However, angiograms with poor signal had already been excluded from the analysis, and older mice generally develop cataracts^82^ which result in a lower transmission of light through the lens, and therefore, a poorer image quality. As tortuosity is a 3-D phenomenon, accurate measurement would require strong signal within single-pixel-deep *en face* images. In this study, a shorter wavelength was chosen to increase the resolution. However, the shorter the wavelength used for OCT, the more attenuating a cataract becomes. Therein lies a trade-off between the axial resolution of the OCT images (the shorter the wavelength is, the higher the resolution is) and the maximum SNR which can be achieved in the retinal images. Although care was taken to apply eye drops rigorously throughout the experiments to ensure cataracts did not form while the animals were under anesthesia,^83^ Three-dimenstional tortuosity measurements would likely be easier to perform at longer wavelengths.

The disorganization of the OPL/ONL structure observed in three transgenic mice and three wild-type mice was not expected from current literature regarding this mouse model. Previous studies in both the mouse^84^ and the human^85^ have attributed similar OPL/ONL splitting to mutations in the CACNA1F gene encoding for the L-type calcium channel ${\mathrm{Ca}}_{v}1.4$ which is also expressed in the ONL of the mouse retina. Without the ${\mathrm{Ca}}_{v}1.4$ calcium channel, photoreceptor synapses are lost, and the dendritic sprouting which occurs in the photoreceptor layer (in the second-order neurons) is abnormal.^86^ Whether this gene is defect in this particular APP/PS1 mouse lineage is a topic which must be explored further.

Regarding the analysis of the immunolabeled wholemounted retinas, strong fluorescent signal appears to derive from a range of sources. Examples of fluorescent signal caused by aggregates of the secondary antibody which adhere nonspecifically to sticky remnants of the vitreous on the surface of the GCL of the retinal wholemounts can be seen in Figs. 9(g)–9(h) and 9(j)–9(k). With the employed immunostaining protocol, non-Aβ specific signal also derives from binding of the anti-mouse secondary antibody to endogenous IgGs present within, e.g., serum and microglia. This makes it difficult to unequivocally assign biochemical Aβ specificity to highlighted structures. Therefore, in order to better delineate potential deposits of intra- or extracellular Aβ, the morphology of the fluorescent signal was also considered. In this study, both retinas of only one transgenic mouse were highlighted as containing candidates for retinal Aβ deposits, displaying intense fluorescence signal, and also presenting with a fibril-accumulation-like structure. Of note, Aβ immunopositive assemblies with similar and distinct fibrillary appearance and a size in the few μm range have been described in human AD retinas.^12^

Previous studies have indicated that Aβ accumulations in the brain are visible with PS-OCT and regular intensity-based OCT,^59^^,^^60^^,^^63^ however, this study was not able to recreate these findings in the retina. This could be due to the difference in size of the Aβ plaques—the plaque candidates that are proposed in Fig. 10 are much smaller than those in the brain [example in Fig. 11(a)]. It has also been previously concluded that not all plaques are visible by either contrast modality.^60^ Hence negative OCT findings do not rule out the presence of retinal Aβ plaques. Even if plaques could be seen in the retina with OCT, it would be difficult to distinguish them from the HRF and the depolarizing deposits that are already present in these retinas as observed in Figs. 5 and 7. In our immunofluorescence protocol, our “positive control” is the brain, and not the retina, as no retinal positive control sample exists. Despite our best efforts to mimic retinal wholemount conditions in the control samples, the tissues are simply not the same, and therefore, it cannot be ruled out that the immunostaining protocol is less optimal for the retina than it is for the brain. However, the candidates for retinal Aβ identified in Fig. 10 would provide an argument that the protocol is indeed suitable.

Given the lack of differences between transgenic and control groups in the *in vivo* OCT data, and the fact that Aβ could only be identified *ex vivo* in 1 out of 11 transgenic animals, the suitability of this APP/PS1 mouse as a model of the human must be called into question where the retina is concerned. As with any other mouse model of AD, it only models some aspects of the disease and not others, and each mouse model will experience different age-related and strain-related changes in addition to anything caused by gene mutation. This study finds itself among the conflicting reports regarding the presence of Aβ in the retina.^8^ Our results indicate that extracellular Aβ may be found in the retina of this mouse model, although not in all, or even most, samples. A much larger study would need to be conducted in order to statistically determine the likelihood of identifying Aβ plaques in the retina of this mouse model. A topic of future exploration could be a comparison of retinal observations in different APP/PS1 mouse models, also adding a quantification of microglia to the retinal analysis.^87^

The cortical Aβ plaque load, evaluated alongside the retinal data, showed a statistically significant increase with age in the transgenic mice. Despite not being able to correlate this to any retinal changes, this study has documented the typical observations that could be expected to be found in the retina of this mouse model, both *in vivo* and *ex vivo*. The 1:1 mapping of the OCT data to the retinal histology was crucial for this experiment, allowing a detailed quantitative and qualitative analysis of structural and functional features of this APP/PS1 mouse model. Tri-fold OCT imaging contrast coupled with retinal wholemounts is, therefore, a promising method for the analysis of animal models of many retinal diseases.

## 5 Conclusion

Although the cortical immunohistochemical staining revealed clear, marked differences in Aβ plaque load between APP/PS1 transgenic mice and their wild-type littermates, a similar difference was not observed in the retina. Candidates for retinal Aβ were only identified in 1 out of 11 transgenic mice. Multicontrast OCT did, however, reveal retinal abnormalities in these mice, including deposits of migrated melanin and a double-banding of the ONL. Owing to the occurrences in both transgenic and control mice, it is likely that these are strain-dependent and not due to the genetic mutation itself. Nevertheless, the combination of multicontrast OCT with 1:1 mapping of retinal histology allowed for a thorough documentation of what one would expect to see in this APP/PS1 mouse model of AD.
